# Identification of Potential Growth-Related Proteins in Chick Vitreous during Emmetropization Using SWATH-MS and Targeted-Based Proteomics (MRMHR)

**DOI:** 10.3390/ijms251910644

**Published:** 2024-10-03

**Authors:** Jimmy Ka-Wai Cheung, King-Kit Li, Lei Zhou, Chi-Ho To, Thomas Chuen Lam

**Affiliations:** 1 Centre for Myopia Research, School of Optometry, The Hong Kong Polytechnic University, Hong Kong; kawai.cheung@cevr.hk (J.K.-W.C.); kk.li@polyu.edu.hk (K.-K.L.); lei.henry.zhou@polyu.edu.hk (L.Z.); chi-ho.to@polyu.edu.hk (C.-H.T.); 2 Centre for Eye and Vision Research (CEVR), 17W, Hong Kong Science Park, Hong Kong; 3 Research Centre for SHARP Vision (RCSV), The Hong Kong Polytechnic University, Hong Kong

**Keywords:** vitreous, emmetropization, SWATH-MS, quantitative proteomics, MRMHR

## Abstract

The vitreous humor (VH) is a transparent gelatin-like substance that occupies two-thirds of the eyeball and undergoes the most significant changes during eye elongation. Quantitative proteomics on the normal growth period in the VH could provide new insights into understanding its progression mechanism in the early stages of myopia. In this study, a data-independent acquisition (SWATH-MS) was combined with targeted LC-ESI-MS/MS to identify and quantify the relative protein changes in the vitreous during the normal growth period (4, 7, 14, 21 and 28 days old) in the chick model. Chicks were raised under normal growing conditions (12/12 h Dark/light cycle) for 28 days, where ocular measurements, including refractive and biometric measurements, were performed on days 4 (baseline), 7, 14, 21 and 28 (*n* = 6 chicks at each time point). Extracted vitreous proteins from individual animals were digested and pooled into a left eye pool and a right pool at each time point for protein analysis. The vitreous proteome for chicks was generated using an information-dependent acquisition (IDA) method by combining injections from individual time points. Using individual pool samples, SWATH-MS was employed to quantify proteins between each time point. DEPs were subsequently confirmed in separate batches of animals individually on random eyes (*n* = 4) using MRMHR between day 7 and day 14. Refraction and vitreous chamber depth (VCD) were found to be significantly changed (*p* < 0.05, *n* = 6 at each time point) during the period. A comprehensive vitreous protein ion library was built with 1576 non-redundant proteins (22987 distinct peptides) identified at a 1% false discovery rate (FDR). A total of 12 up-regulated and 26 down-regulated proteins were found across all time points compared to day 7 using SWATH-MS. Several DEPs, such as alpha-fetoprotein, the cadherin family group, neurocan, and reelin, involved in structural and growth-related pathways, were validated for the first time using MRMHR under this experimental condition. This study provided the first comprehensive spectral library of the vitreous for chicks during normal growth as well as a list of potential growth-related protein biomarker candidates using SWATH-MS and MRMHR during the emmetropization period.

## 1. Introduction

Myopia is a global concern, as more than 22% of the world’s current population is affected by this condition, making it the most prevalent refractive error worldwide. Furthermore, predictions suggest this number is expected to increase to 50% by 2050 [1]. Although myopia can be corrected by spectacles, contact lenses, or surgery, the rapid increase in its prevalence has raised alarming concerns regarding its association with high myopia (typically defined as −5.00D or −6.00D or below), where eyes with high myopia are more susceptible to sight-threatening ocular diseases, such as retinal detachments and glaucoma, due to the excessive elongation of the eyeball [2,3,4]. The onset of myopia has been suggested to be affected by multiple factors, such as genetics [5] and the environment [6,7]. However, since the fundamental mechanism of eyeball growth is unclear, prevention and control are complex and challenging.

The emmetropization period (normal growth) describes the stage during which the eye elongates gradually from a relatively short axial length (typically at a hyperopic state) to eventually matching the eyes’ optical power to its axial length, resulting in clear and focused vision in unaccommodated eyes. This period is commonly observed in humans [8] as well as various animal species, including monkeys [9], chicks [10], and guinea pigs [11]. Myopia progression can be considered as a failure of emmetropization, where the eyeball further elongates, passing the emmetropization state with an indefinite stop sign as it progresses. For both emmetropization and myopia progression, the vitreous chamber depth (VCD) is the main contributor to the overall change in the axial length of the eyeball. From this accelerated normal growth pattern, proteins responsible for this elongation might bring us more insight into the potential factors related to abnormal eye growth and myopia progression.

Chicks have long been used as an animal model for ocular research [10,12,13,14] due to the ability of their eyes to compensate for the surrounding environment. Just like humans, the eyes are hyperopic at birth, and their refraction shifts towards emmetropia with age [10]. The vitreous humor (VH) is a transparent gelatin-like substance composed of networks of collagen fibrils and hyaluronic acid that takes up to 80% of the eyeball at the posterior part of the eye cavity. The collagen/hyaluronic acid meshwork (gel-like substance) is also ideal as a protein storage reservoir within its close space, allowing soluble proteins within the vitreous metabolic nutrient movements to other parts of the eyeball, including the lens, ciliary body, and retina [15,16,17]. Furthermore, being located adjacent to the retina also enables the transportation of chemicals and proteins via diffusion to maintain homeostasis within the eyeball [18].

Proteomic-based analyses in ocular research have enabled the identification of biomarkers in conditions such as myopia [19,20] and diabetic retinopathy [21,22,23]. However, published proteomic research on the vitreous is still scarce due to the limitation of the traditional gel-based proteomic approach on identifying low abundant proteins within the diluted nature of the vitreous. The demanding sample volume and concentration used in traditional gel-based proteomics significantly limits the detection of proteins in the vitreous, hampering its potential use and scientific research value. Quantitative proteomics offers an additional method to understand protein dynamics by comparing the levels of proteins under different conditions. Sequential window acquisition of all theoretical mass spectra (SWATH-MS) is an information-dependent acquisition (IDA) method that could adjust the poor sensitivity of traditional shotgun proteomics [24] by acquiring all the spectrums from MS/MS without being dependent on the previous ionization threshold of TOFMS [25]. Typically, a 25 Da precursor window or variable size *m*/*z* windows are set to be scanned across a mass range of interest, passing all the ions into the collision cell for the entire liquid chromatography separation. The TOF analyzer will then fragment and analyze the ions. Due to the small windows scanning throughout the spectrum, a very fast scanning time will be needed. With the multiple windows that were applied throughout the mass range, fragmented ions in the given window will be more easily associated with their precursor ion, and more specific MS and MS/MS spectra will be obtained. However, as this is a DIA method, the data acquired from the SWATH-MS mode will require a known database (from the IDA mode) for spectra matching and processing. Therefore, a comprehensive vitreous proteome covering the emmetropization is still required for quantifying proteins using SWATH-MS, allowing a more profound and broader search of these potential growth-related proteins.

Single/multiple-reaction monitoring (MRM) has been an emerging MS-targeted proteomic quantitation approach for validating specific peptides while offering results with high sensitivity and specificity [26,27,28]. The MRM approach targets specific peptides only by selecting the specific precursor mass of the known peptide for tandem MS/MS fragmentation, allowing a list of specific peptides to be identified and quantified if suitable peptides of a protein are isolated (proteins of interest). A predesigned transition list of the peptides of the target protein should be obtained and the first quadruple (Q1) selects the precursor ion of the target peptide. This is followed with the Q2, where it acts as a collision cell, fragmenting the precursor ions. Lastly, the Q3 selects the more specific fragment ions of the target peptide by detecting only the ions with a specific predefined *m*/*z*, ensuring specificity. This allows specific peptides to be identified within the selected time frame, while ignoring all the other peptides for fragmentations, allowing a longer dwell time, and resulting in a higher signal-to-noise ratio (S/N) for more precise differentiation of peaks. While this system usually runs under a microflow (with a larger sample amount required), the size of the columns is usually larger with a higher flow rate.

The relatively new, high-resolution MRM (MRM^HR^) approach with the Triple-TOF MS running under a nanoflow (which is similar to the SRM/MRM approach) was adopted in this study to solidify further our findings of SWATH results of differentially expressed proteins found across the time points for the benefit of low amount of peptide consumption [29]. The multiplexed looped MS/MS acquisition mode allows the targeting of multiple proteins based on the high-resolution capabilities of the TripleTOF 6600 system in MRMHR mode. This is essential for vitreous sample studies to maintain sensitivity [30] since the peptide from an individual sample is very limited.

In this study, we have generated the first and most comprehensive vitreous proteome for chicks covering the normal growth period in the first month after hatching [31]. To identify and validate these differentially expressed proteins (DEPs), including their functions and roles involving the biological pathways, will provide a better understanding of the proteomic changes during emmetropization. Also, these findings may provide insight into the normal growth mechanism from a range of time points (D7, 14, 21 and 28) with proteins that are actively responsive to rapid ocular elongation during different time frames.

## 2. Results

### 2.1. Biometric Measurements of Chicks during Normal Growth

**Figure 1:**
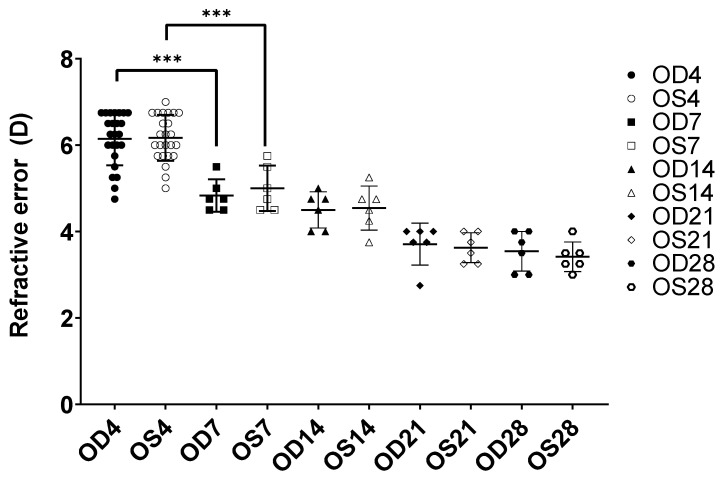
Measured refractive error of the right eye (OD) and left eye (OS) during different time points of normal growth (4, 7, 14, 21 and 28): *n* = 6 at each time point. *** *p* ≤ 0.001; one-way ANOVA. No significant differences were found between OD and OS at all time points.

Ocular refractive and biometric measurements were measured on days 4, 7, 14 and 28. Six normal-growing chicks were raised at each time point (a total of 24 chicks throughout this study). The baseline measurements were measured on day 4 to ensure the eyes grew normally. No significant differences in terms of biometric measurements were found between the left (OS) and right eyes (OD) (*p* > 0.05, paired *t*-test) at all time points and the refractive error measurements are shown in Figure 1.

**Figure 2:**
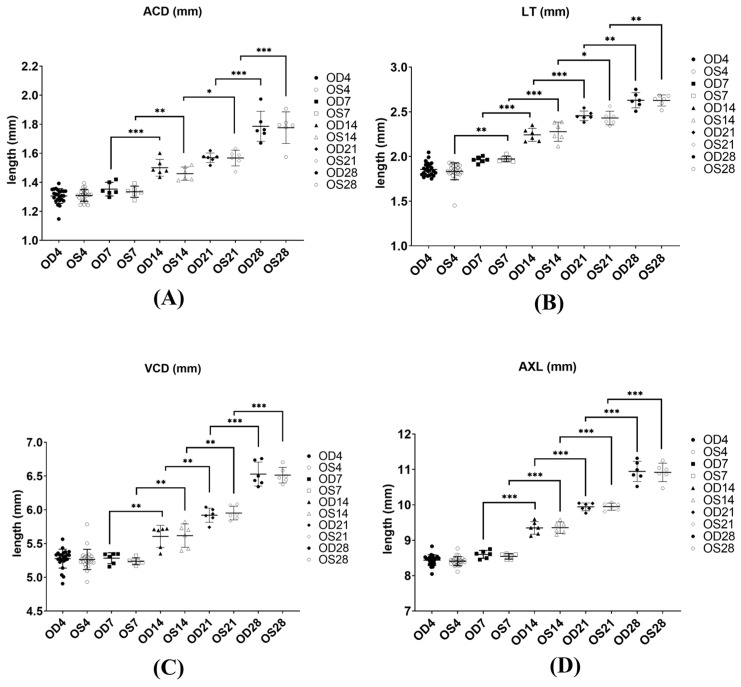
Length of ocular components of the right eye (OD) and left eye (OS) during different time points (4, 7, 14, 21 and 28) of normal growth: (**A**) ACD: anterior chamber depth; (**B**) LT: lens thickness; (**C**) VCD: vitreous chamber depth; (**D**) AXL: from the front of the cornea to the front of the retina; *n* = 6 at each time point. * *p* ≤ 0.05, ** *p* ≤ 0.01 *** *p* ≤ 0.001, one-way ANOVA. No significant differences were found between OD and OS at all time points for ACD, LT, VCD and AXL.

In terms of refraction, all eyes measured on baseline (day 4) were hyperopic (6.15D ± 0.61 for OD and 6.17D ± 0.52 for the OS). The most significant differences in refractive error were found only between D4 and D7 for both the right and left eyes. In terms of ocular parameter lengths, the anterior chamber depth (ACD), lens thickness (LT), vitreous chamber depth (VCD) and axial length (AXL) increased significantly during the growth period mostly from D7 onwards for both eyes (OD and OS, *p* ≤ 0.05, one-way ANOVA). The average changes of various ocular parameters measured from an A-Scan are shown in Figure 2A–D.

**Figure 3:**
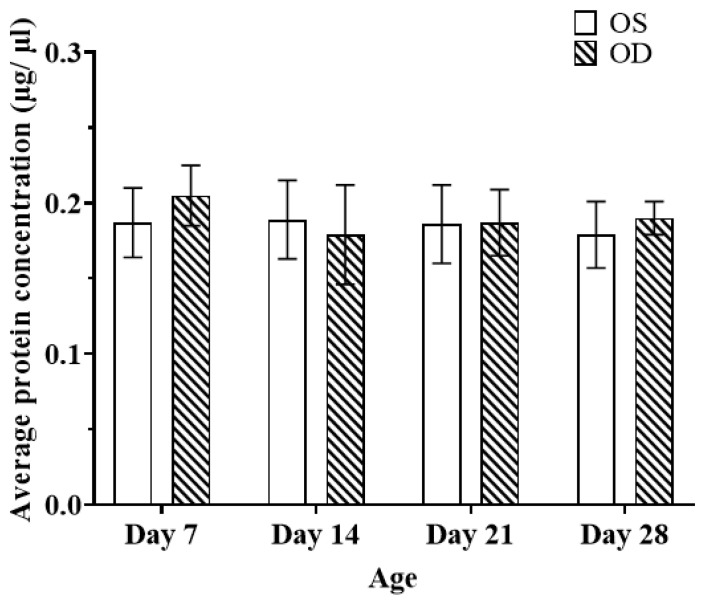
Average protein concentration of the homogenized vitreous, *n* = 6 at each time point. Paired *t*-test between eyes and one-way ANOVA for multiple time points.

There were no significant changes in the total protein concentrations across all time points (Figure 3). An average of 0.187 ± 0.023 μg/μL and 0.205 ± 0.020 μg/μL for OS and OD were observed on day 7, respectively. For day 14, an average of 0.189 ± 0.026 μg/μL and 0.179 ± 0.033 μg/μL for OS and OD were observed, respectively. For day 21, an average of 0.186 ± 0.026 μg/μL and 0.187 ± 0.022 μg/μL for OS and OD were observed, respectively. Lastly, for day 28, an average of 0.179 ± 0.022 μg/μL and 0.190 ± 0.011 μg/μL for OS and OD was observed, respectively.

### 2.2. Generation of the Normal Growth Chick Vitreous Proteome

**Figure 4:**
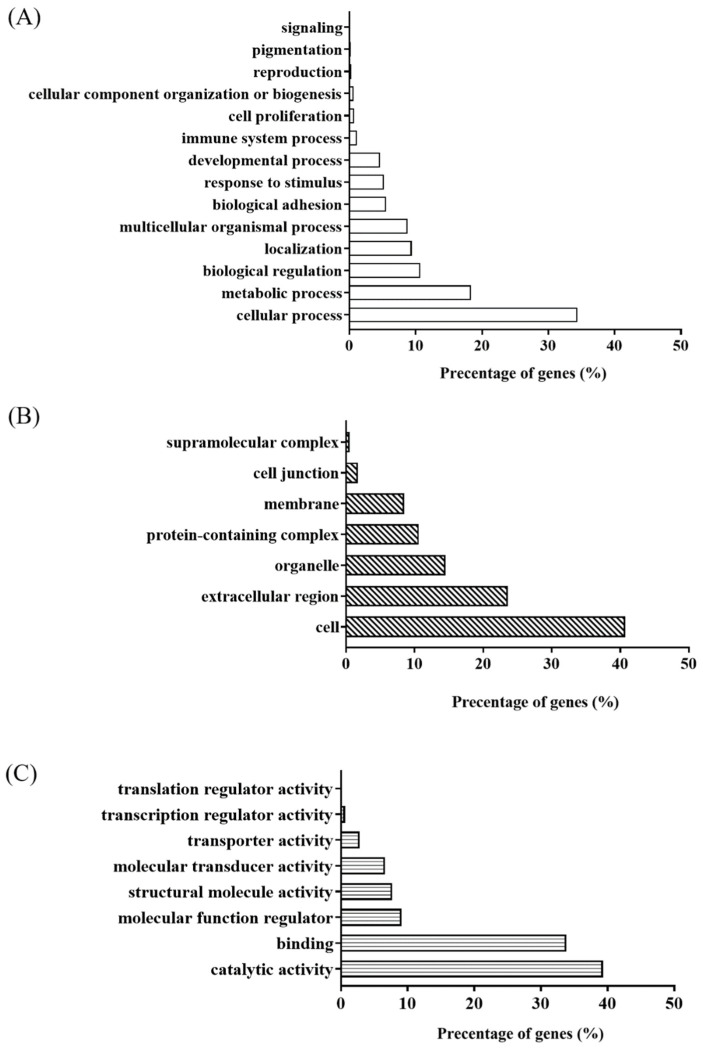
Gene ontology (GO) classifications of proteins from combined search proteins of normal growing chick vitreous. (**A**) Biological process. (**B**) Molecular function. (**C**) Cellular components using PANTHER.

A total of 1576 non-redundant proteins (22987 distinct peptides) were identified from a combined search using 8 information dependent acquisitions (IDA) consisting of all eyes from the four time points at a 1% FDR, the full list of proteins can be found in Supplementary Table S1. These identified proteins at a 1% FDR were loaded onto the PANTHERTM online gene ontology system 34 for the global overview of the gene ontology (GO) functions on the normal vitreous proteome for chicks. A total of 935 ID proteins (60% of all the identified proteins) were successfully mapped, and their classification (biological process, molecular functions, and cellular components) is shown in Figure 4. For biological process (Figure 4A), the top three processes were found to be as follows: cellular process (GO:0009987) 34%, metabolic process (GO:0008152) 18%, and biological regulation (GO:0065007) 11%. For molecular functions (Figure 4B), the top three functions were as follows: catalytic activity (GO:0003824) 40%, binding (GO:0005488) 34%, and molecular function regulator (GO:0098772) 9%, In terms of the cellular component (Figure 4C), the top three leading portions were as follows: cell (GO:0005623) 41%, extracellular region (GO:0005576) 23.6% and organelle (GO:0043226) 15%. The proteins identified in this study were compared to our previous vitreous study on chicks using ICPL labeling [20], having an increase of around 13x more proteins to be identified, while 159 proteins were found as “core proteins”.

### 2.3. Ion Library Generation and Quantitation for SWATH-MS

**Figure 5:**
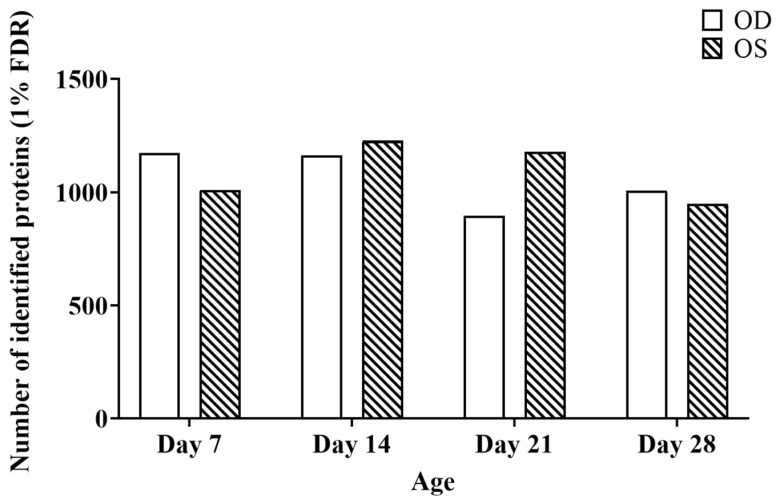
The number of proteins identified at a 1% FDR at each time point for Left (OS) and Right (OD) pool.

The number of protein identifications (at a 1% FDR) from each time point for each eye is shown in Figure 5. A merged ion library consisting of unique proteins from these eight IDAs from all experimental groups (OD and OS groups at each time point) was generated (shown in Figure 5). Although a total of 1988 unique proteins were identified: under the IDA mode, only 542 (27%) proteins were commonly found across all the samples to allow quantitation. However, although there were only 1576 total proteins found from the combined generated ion library for SWATH-MS, a total of 1456 (92%) common proteins could be acquired across all the samples using this method.

### 2.4. Protein Quantitation of Chick Vitreous Proteins during Normal Growth Using SWATH-MS

**Figure 6:**
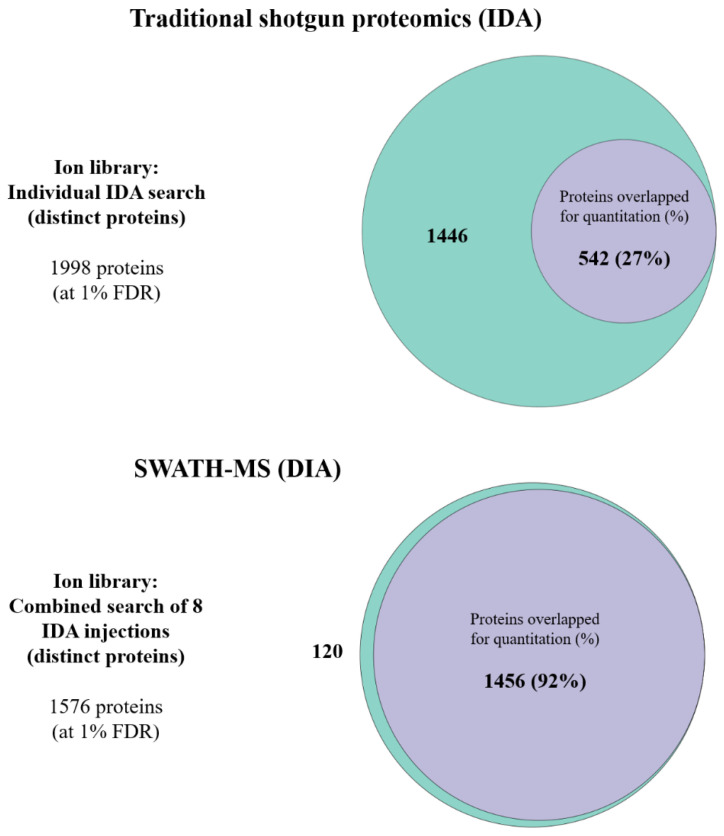
Comparison between the combined library and 8 individual libraries; Traditional shotgun proteomics (8 individual injections, individually searched) and combined searched library (8 injections combinedly searched). The total unique proteins (peptides) identified from all 8 individual injections, and a combined search were 1988 and 1576, respectively. The total overlapping unique proteins found across all 8 injections in IDA and DIA mode were 542 and 1456, respectively. The purple circle indicates the number of proteins that were overlapped for quantitation. The green circle shows the number of distinct proteins that remained.

As more common proteins could be detected using the SWATH-MS approach, we aimed to investigate the aging effect on protein profiles during the normal growth period in the chick vitreous using this DIA quantification (Figure 6). A total of 24 SWATH injections (OD and OS at each time point, with three technical replicates) were integrated and processed using PeakView software and the results were exported for statistical calculation.

**Figure 7:**
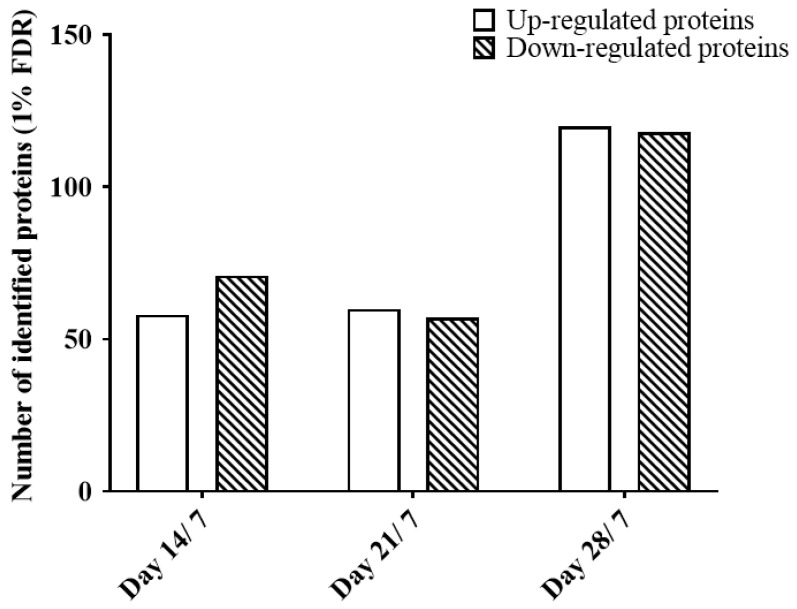
The number of proteins that are found differentially expressed from each time point using the combined ion library. The FC ratio was calculated by comparing it to day 7. Fold change calculation filter was set as ≥1.5 FC, same direction for both eyes.

Using a filter criterion listed in the method section for the fold change (FC) (≥1.5 FC, at least two peptides per protein, and the FC must be in the same direction for both eyes), compared to day 7 (Figure 7): 58 up-regulated and 71 down-regulated proteins were found in the D14 group (D17/D7). A total of 60 up-regulated and 54 down-regulated proteins were found in the D21 group (D21/D7). Moreover, a total of 120 up-regulated and 117 down-regulated proteins were found in the D28 group (D28/D7). A table of all the overlapped DEPs identified across all the time points can be found in Supplementary Table S2. All raw data generated from the information dependent (IDA) and SWATH acquisitions (DIA) were accepted and published in the Peptide Atlas public repository (http://www.peptideatlas.org/PASS/PASS01258, accessed on 27 September 2019) for public open access [31].

**Figure 8:**
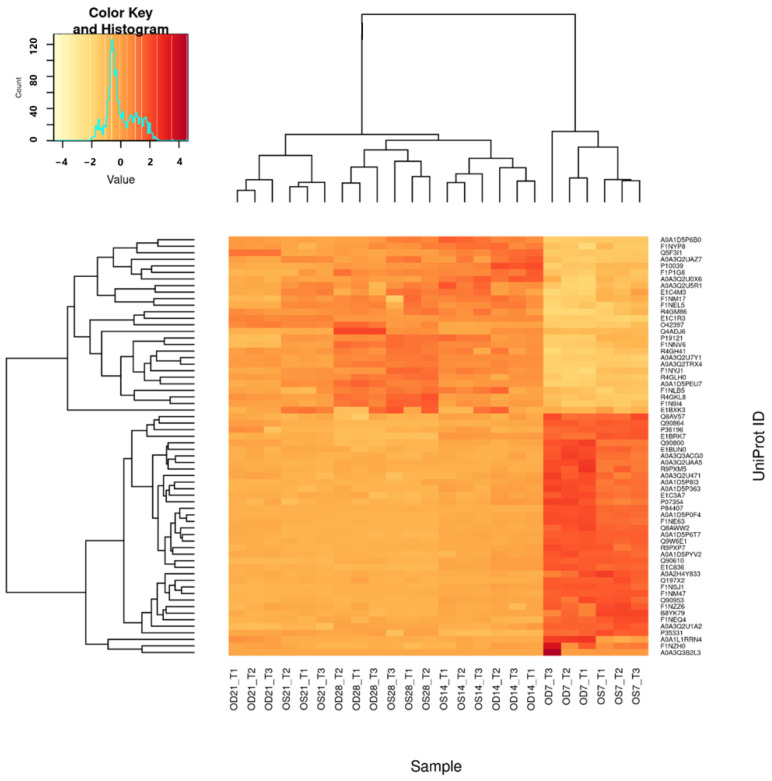
Heatmap showing DEPs in normal eye growth period (days 7, 14, 21 and 28). Dark red indicates a higher intensity value, and yellow indicates a lower intensity value. For upregulated proteins, 27 Proteins had low intensity on day 7 (yellow) and remained high for the rest of the time points. While 37 proteins had a high intensity on day 7 (dark red) and remained low for the rest of the time points.

Among all the DEPs found from all time points, 38 proteins (12 up-regulated and 26 down-regulated proteins) were commonly identified across all time points. The dynamic changes in these could be critical in the normal growth process. These were then submitted to draw clustered heatmaps using the HeatmapMaker in R program (Juan Pablo Carreón Hidalgo), shown in Figure 8. The expression level for each protein was calculated using the formula: [(total area of each protein) − mean/SD]. Dark red indicates a more positive value (i.e., up-regulated proteins) and yellow indicates a more negative value (i.e., down-regulated proteins). Among these, potential normal growth-related DEPs are listed in Table 1.

### 2.5. Data Analysis Using Bioinformatics Software and Confirmation Using Targeted Proteomics

**Figure 9:**
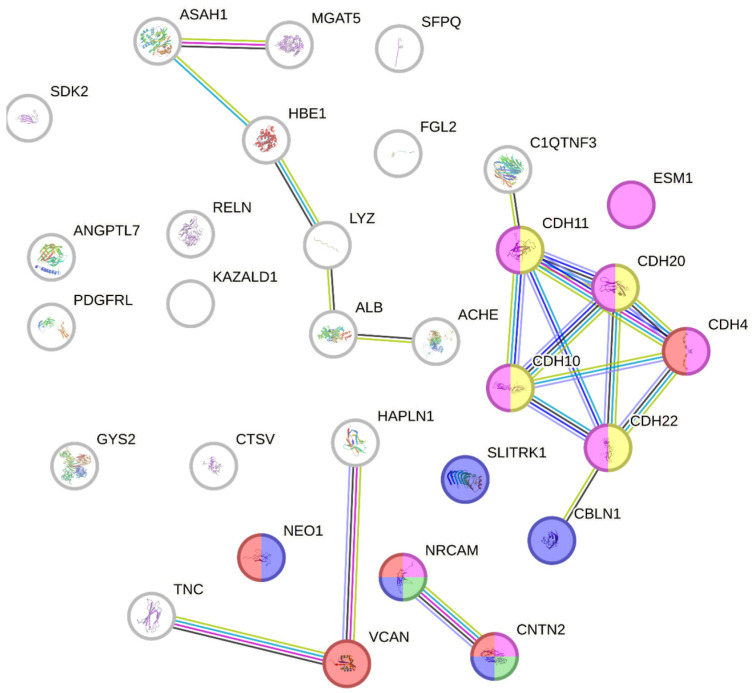
STRING analysis on common differentially expressed proteins (DEPs) across all the time points (total of 38 proteins). The color represents: Red: cell adhesion molecules; Green: cell–cell adhesion and anchored component of synaptic membrane, Pink: cell adhesion molecule binding, Yellow: cell–cell junction assembly, Blue: axon guidance and intrinsic component of synaptic membrane.

As we intended to screen for proteins with temporal changes during emmetropization, commonly found differentially expressed proteins (a total of 38 proteins) were loaded on the STRING database for protein–protein interaction network analysis (Figure 9). Several proteins were grouped and found to be responsible for cell adhesion, including VCAN, NEO1, NRCAM, CNTN2, RELN and the cadherin family group. Meanwhile, VCAN, NRCAM and CNTN2 were also responsible specifically for neuron cell–cell adhesion. Most of the DEPs found were involved in the cell-adhesion molecules pathway (with an FDR of 3.72 × 10^−7^)

### 2.6. Protein Validation Using High-Resolution MRM (MRM^HR^)

**Figure 10:**
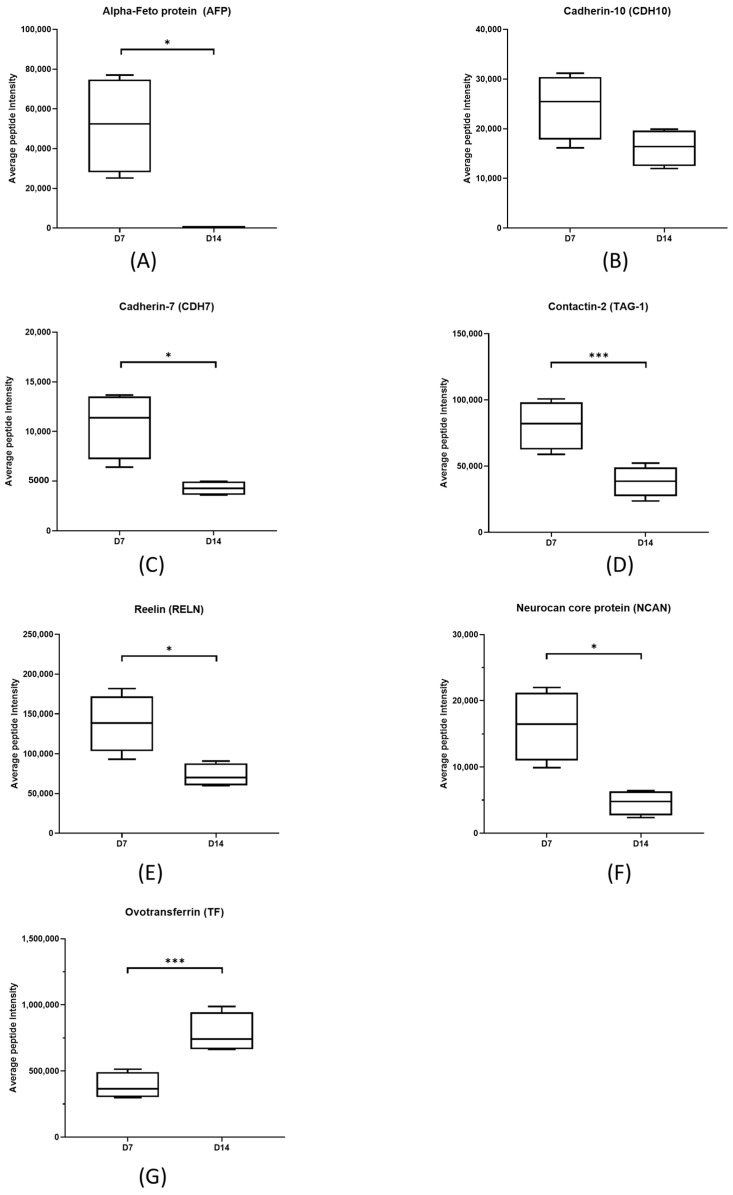
The fold change of DEPs (**A**–**G**) obtained from SWATH-MS (Study 1) was validated using MRMHR in Study 2 (D7 and D14) from individual vitreous samples from random eyes collected in separate batches of chicks (*n* = 4 at each time point), * *p* < 0.05; *** *p* < 0.01, unpaired *t*-test. Error bars = Mean ± SD. The top 3 transitions were averaged and the top 3 peptides were averaged as the average peptide intensity of each protein. The intensity was normalized using GAPDH and the fold change was calculated by dividing D14 by D7.

To further validate the expressions of these key proteins, day 7 and 14 time points were selected due to the highest magnitude of the FC (either up-regulated or down-regulated) observed during proteomic analysis, shown in Figure 9. A separate batch of chicks (*n* = 4 at each time point) on days 7 and 14 was raised with the same conditions for validation of protein expressions using a high-resolution multiple-reaction monitoring (MRM^HR^) targeted proteomics strategy. No significant difference was found in GAPDH expression using both the SWATH-MS and MRM^HR^ approaches; therefore, GAPDH was used as the internal standard to normalize all the samples for each experiment for calculation. A total of seven differentially expressed proteins (ovotransferrin, reelin, contactin-2, alpha-fetoprotein, cadherin-7, cadherin-10, and neurocan core protein) were successfully confirmed using MRM^HR^ with similar FC (day 14/day 7) found from SWATH-MS study (*p* ≤ 0.05, shown in Figure 10). The average peptide ratio was calculated from the top three transitions from three peptides of the protein and normalized with GAPDH. A full transition list of the peptides of each protein can be found in Supplementary Table S3. Unlisted proteins were not detectable in the MRM^HR^ validation study ion library and, therefore, were excluded.

## 3. Discussion

As the change in the vitreous chamber depth (VCD) is often the result of axial elongation during emmetropization, this study is the first to apply a next-generation proteomics approach (SWATH-MS) to investigate the underlying mechanism of emmetropization in the chick vitreous. Early gel-based vitreous proteomic studies were mainly limited by the inability to detect low-abundance proteins due to the high concentration of several proteins, such as albumin and transferrin. Even in fractionation techniques like immune depletion, the identification rate of low-abundance proteins in gel-based studies was low [22]. Advancements in MS technologies, including improved sensitivity and resolution, have benefited vitreous-related studies as the low protein content can now be further identified. Recent proteomic studies on various vitreous-related ocular diseases, such as AMD [32], DR [23], and myopia [20], have allowed the acquisition and identification of more proteins within the vitreous, expanding their applications in ocular diagnostics. Regarding growth studies, Yee et al. identified 1217 proteins, with 43 proteins showing differential expression, in a study comparing the embryonic and young adult vitreous in humans [33]. Apart from human studies, the proteomic analysis of the vitreous was also studied in various animal models including the rabbit model, where Liu et al. compared the vitreous proteins in young and mature rabbits, identifying a total of 466 proteins [34]. In a mouse model study, a total of 626 proteins were identified between the vitreous and retina [35]. As well, in an LIM study, an ICPL labeling approach was used to quantify vitreous proteins in lens-induced chicks, where APOA-1 and purpurin were successfully quantified and validated [20]. Within these proteomes from animal studies, several top conserved proteins, including albumin (ALB), adenylate kinase isoenzyme 1 (AK1), beta-crystallin (CRYBB), vimentin (VIM) and fructose-bisphosphate aldolase (ALDOC), were found within the human study mentioned earlier, indicating that the use of an animal model could provide further valuable insights in vitreous development relating to human ocular biology.

In this study, the overall changes in refraction and ocular parameters (AXL) indicated normal eye growth, which was consistent with previous findings in a study of chicks [10]. This finding showed a reduction in diopters (D) after hatching, confirming the emmetropization period. The average changes in refraction showed a reduction in diopter (D) from a hyperopic state on day 7 to near normal on day 28, where no significant difference was found between both eyes at each time point (≥ 0.05, paired *t*-test). Due to the limited vitreous volume obtained from chicks, lysed vitreous samples from six individual chicks of the same age were combined, pooling an equal number of proteins from each eye as a pool of vitreous samples (left and right eye POOL at each time point). This pooling approach was similar to our previous vitreous study design [20]. To minimize the chances of false-positive findings, stringent filters were applied to the results: Separate injections were acquired on the left and right eye samples, and the quantified proteins needed to have the same FC direction and an FC ≥ 1.5-fold. Using a highly sensitive nanoLC-ESI-MS/MS system, this study identified 1576 non-redundant proteins (22,987 distinct peptides) at a 1% FDR without fractionation or protein depletion. This makes it the most comprehensive vitreous protein library for chicks to date, covering the emmetropization period from day 7 to day 28, expanding on our previous study using the chick model [20]. The top abundance proteins identified were serum albumin, reelin, fibronectin, tenascin, and ovotransferrin. A GO analysis of this data set revealed similarities in the molecular functions, biological processes and cellular components compared to vitreous proteome studies in humans [18].

The use of SWATH-MS to quantify vitreous proteins significantly enhanced the detection capabilities and repeatability, particularly for those lower abundant proteins [36]. In this study, the SWATH-MS approach acquired more than 92% of the proteins across all the sample injections, whereas only 29% of proteins were found across all the samples using the conventional IDA-based approach. This demonstrates that the use of a SWATH-MS in the chick vitreous is highly advantageous compared to the traditional IDA method, as it provides a larger pool of vitreous protein candidates to be quantified. Applying the strict filter criteria mentioned, the number of DEPs was similar in the first two time points (D14 and D21) but doubled in the last time point (D28/7), as shown in Figure 8. This was expected, as the greatest physical changes occurred near the end of the holding period (Figure 3) compared to the baseline. DEPs that were quantified across all the time points were further analyzed to determine their functions possibly related to normal growth/emmetropization. The degree of FC of these proteins was greatest at the first time point (D14/D7) and remained similar at later time points, indicating a possible role/function during the growth period (Figure 9).

Single reaction monitoring or multiple reaction monitoring (SRM/MRM) offers higher sensitivity and selectivity compared to traditional identification method [37], where specific peptide transitions will be monitored and compared to its control. The high-resolution MRM (MRMHR) approach was applied to solidify our findings of the SWATH results of differentially expressed proteins across the time points further. A separate batch of chicks (*n* = 4) was raised under the same conditions. The vitreous was collected on days 7 and 14 for protein validation using MRMHR. Both the SWATH-MS and MRMHR datasets showed similar directionality and fold changes for the differentially expressed proteins. The DEPs quantified primarily belonged to the categories of structural and neuronal proteins relating to the growth period, which can be further categorized into several groups, as detailed below.

### 3.1. Alpha-Fetoprotein (AFP)

Alpha-fetoprotein is one of the earliest serum glycoproteins synthesized by the liver. It is present in the fetal yolk sac, cerebrospinal fluid, amniotic fluid, and the vitreous body [38] during embryo and fetal development. The level of AFP rapidly declines to less than 10 ng/mL as it reaches adulthood in humans [39]. AFP shares similarities in molecular weight and structure to albumin and was found to be an early form of albumin [40]. Its role in development involves serving as a carrier protein for various substances, such as binding to fatty acids and bilirubin, for a transportation role during early development [41]. It has been identified in human fetal vitreous, peaking at week 17 and slowly declining towards 24 weeks of age [42]. Similar patterns have been observed across species, such as rats [43,44], where AFP levels rise rapidly during fetal life and then decrease shortly after birth.

### 3.2. Cadherin Superfamily Group

CDH4, CDH7, CDH8, CDH10, CDH11, CDH20, and CDH22 were found to be downregulated on days 14, 21, and 28 compared to day 7 from this study using SWATH-MS. Among these, CDH7 and CDH10 were successfully confirmed using MRMHR in a separate batch of chicks. Cadherins are cell surface adhesion glycoproteins that play a primary role in tissue and organ development [45]. They are Ca+ dependent trans-membrane structural proteins with a primary function of cell–cell adhesion during cellular growth, cell polarization, and differentiation [46]. As a structural protein, cadherin is essential for maintaining cell integrity. In the vitreous, they localize to focal adhesions and promote adhesion to fibronectin, which provides structural support throughout the vitreous. CDH10 and CDH7 belong to the type II classical cadherins within the cadherin superfamily. These cadherins are usually found in specific brain regions or circuits such as cerebellar, retinal, and hippocampal circuits. Cadherin-10 has been discovered in restricted brain regions and neural retinas [47]. Its expression level was found to increase in the embryonic nervous system of embryonic zebrafish [47] and chicken brain [48]. Cadherin-7 is encoded by the CDH7 gene and is involved in the development of the vertebrate nervous system and expressed in the early phase of cranial motoneuron development (during axon extension) [49].

### 3.3. Contactin-2 (TAG-1)

Contactin-2 (TAG-1) is a neural cell adhesion molecule that belongs to the immunoglobulin superfamily (IgSF). It consists of six immunoglobulin-like domains and four fibronectin repeats [50,51]. TAG-1 plays a crucial role in axon extension, growth cone guidance, and myelination during development [52,53]. During the fetal period, it aids in forming axon connections in developing nervous systems by guiding development on the axon surface [50]. TAG-1 has a high affinity to the neural cell adhesion molecule (NCAM) and acts as a neural cell adhesion molecule ligand for binding to the neurocan for cell–cell interactions during nervous tissue histogenesis in chickens [54].

### 3.4. Neurocan Core Protein (NCAM)

The neurocan core protein (NCAM) is a brain chondroitin sulfate proteoglycan (CSPG) that is synthesized by neurons [55]. It belongs to the lectican family, which includes other members such as aggrecan, versican and brevican. These proteins play roles in cell–cell interactions and nervous tissue histogenesis as a member of this family, NCAM exhibits the ability to bind to hyaluronan. It is one of the most abundant CSPGs during brain development and is primarily involved in neuron guidance and modulation of cell adhesion and migration during normal development [56,57,58]. The NCAM has been detected in the retinal layers of embryonic rat retina but not in later stages [59,60]. In rat brains, NCAM levels peak during late embryogenesis and gradually decrease after the first month after birth [61], possibly due to the proteolytic processing of the NCAM reducing its level.

### 3.5. Reelin (RELN)

Reelin (RELN) is a large extracellular glycoprotein expressed highly in the brain. It plays crucial roles in neural cell positioning, neuronal migration [62], growth cone guidance [63], and synaptic plasticity [64]. RELN regulates microtubule functions and neuronal migration during the development of the brain by binding to the ApoER2 receptor [65]. It is essential for the normal development of cortical, hippocampal and cerebellar neuronal lamination [66]. RELN binds to its receptors apolipoprotein E receptor 2 (ApoER2) and very-low-density lipoprotein receptor (VLDLR), leading to the Src family tyrosine kinase (SFK)-mediated tyrosine phosphorylation of disabled-1 (Dab 1) in the reelin signaling pathway [65]. The downstream activation of SFK is vital for regulating various cellular functions, including cell proliferation, differentiation and metabolism [67].

### 3.6. Ovotransferrin (TF)

Ovotransferrin (TF) belongs to the transferrin family, consisting of iron-binding glycoproteins [68]. These proteins primarily participate in iron metabolism in tissues by transferring ferric ions. TF has been suggested to possess an anti-oxidative [69] effect and the up-regulation of ovotransferrin might indicate the presence of oxidative stress during the elongation of the eyeball. The TF gene and protein from choroid could be a regulator of myopic eye growth [70], as evidence showed an increase in mRNA levels in the recovering retina, retinal pigment epithelium (RPE) and choroid of a chick form-deprivation myopia (FDM) model. Additionally, up-regulation of TF has been detected in the chick vitreous after LIM treatment [20], further suggesting that TF could be an early indication of ocular elongation or myopia progression.

Although the changes of these proteins were not previously reported in the developmental process in the chick vitreous, similar changes in the expressions of these proteins during tissue development from other studies have suggested potential roles for these MS-confirmed molecules in emmetropization eye growth. In addition to proteins responsible for structural properties, several neuronal-specific proteins were also identified, further suggesting that the vitreous could serve as a compartment for biofluid leakages from surrounding locations such as the retina and brain. This highlights the potential of the vitreous to be a potential collection or diagnostic site for brain-related proteins. Since the vitreous can be easily extracted during routine vitrectomy, it may offer as an alternative substance for brain-related diseases diagnostic.

The down-regulation of these cell–cell adhesion proteins might indicate the elongation and stretching of the vitreous gel structure or that these structural proteins are no longer needed once the axon growth is complete. Reelin functions downstream of TGF-β1, and the opposite expression found in this study might indicate an increased level of TGF-β, which is associated with myopia in remodeling the sclera during myopia development [71,72,73]. The down-regulation of these cell adhesions, axon guidance, and extracellular matrix proteins suggested that the vitreous was slowly stabilizing and reducing the need for growth signals. This overall pattern suggested a shift from a more dynamic, growth-orientated phase (day 7) to a more stabilized phase (day 28), while some angiogenesis and proliferation-related functions are still needed (the upregulation of ESM1 and TEFW). With these DEPs identified during the emmetropization period, it would be beneficial to observe their functions and roles closely, as well as conduct functional studies under myopic conditions. These protein changes demonstrate that the vitreous is not simply a clear, transparent gel tissue providing structure stability, but it also exhibits dynamic protein exchanges within this material, expanding its potential as a tissue for reporting the ocular condition. Although the SWATH-MS filter setting in this study was applied to reduce false-positive results, other DEPs found (not across all time points) are also of interest, and further validation steps should be taken. One potential weakness of this study is the pooling of chick vitreous samples due to the low concentration of proteins, which may underestimate the actual changes observed during the conditions. However, this issue was mitigated by applying strict filters, where the fold change must be in the same direction and pass the cut-off. Fractionation of the vitreous samples could increase the number of proteins found in the vitreous [18] to extend the coverage of the ion library. Furthermore, only two time points were used for MRMHR validation due to the greatest fold change observed during those time points; it would be ideal to include the remaining time points in future studies.

## 4. Materials and Methods

### 4.1. Data Measurements and Vitreous Sample Collection

A total of 24 (6 for each time point) White Leghorn chicks (Gallus gallus domesticus) were used in this study. Chicks hatched from specific pathogen-free (SPF) eggs were housed in stainless steel brooders under a 12/12 dark/light cycle. The breeding room had an automatically controlled temperature environment with an average temperature of 25.8 °C and a humidity level of 41.8%. Food and water were given ad libitum. Handling and operations complied with the ARVO statement approved by the Animal Subjects Ethics Sub-committee (ASESC), The Hong Kong Polytechnic University.

### 4.2. Ocular Growth and Weight Measurements

Ocular growth and weight measurements were taken at set time points: days 4 (baseline measurement data), 7, 14, 21, and 28 during the normal growth (emmetropization) process. The refractive error was measured using a streak retinoscope (Beta 200 Streak Retinoscope Set 2.5v, Heine, Germany) with a trail lens bar (±16.00D in 0.5D steps) in dim light conditions. Sphere equivalent (S.E.) measurements were used to define the refractive error in this animal study (S.E. Spherical power + ½ cylindrical power). An A-Scan Ultrasound (5073PR, Olympus, Toyoko, Japan) coupled with a 30 MHz probe (PZ25-025-R1.00, Panametrics, MA, USA) was used to measure ocular length. A lid retractor was used to keep the eye open during measurements, and anesthesia was not applied to avoid any potential protein changes induced. An average of 3 repeats of measurements were performed on each eye for analysis. Ocular components, including the anterior chamber depth (ACD), lens thickness (LT), vitreous chamber depth (VCD) and axial length (AXL), were analyzed and compared in this study. The weight of each chick was measured using an electronic balance (200 M, Precisa, Switzerland) after the A-scan and refractive measurements. Day 4 was considered the baseline measurement for proteomic analysis and ocular samples were collected on days 7, 14, 21 and 28.

Chicks were sacrificed with CO_2_ overdose, and the optic nerve was cut immediately to isolate the eyeball. The eyeball was then kept and washed with ice-cold phosphate-buffered saline (PBS). The eyeball was then hemisected equatorially using a razor blade, cutting the eyeball into the anterior and posterior parts, exposing the vitreous. The vitreous was then pushed out without damaging the retina layer using a pair of tweezers. The main body of the vitreous was extracted alongside the pecten oculi inside. After removing the main vitreous body, remains of the liquid vitreous were also collected using a pipette and immediately put into a 1.7 mL tube (Eppendorf, Hamburg, Germany) to be weighed and kept in liquid nitrogen during the dissection period. Collected samples were transferred to −80 °C for sample storage until further use after the collection period. It was made sure that there was no evidence of blood or tissue contamination in the vitreous at the time of sample harvesting at all the time points.

### 4.3. Vitreous Sample Preparation

Details of the sample preparation are described in our previous publication [31]. Frozen vitreous samples were removed from −80 °C and a 1:1 *w*/*v* (vitreous sample: lysis buffer) ratio of tissue protein extraction reagent (T-PER, Cat# 78510, Thermo Fisher Scientific, Waltham, MA, USA) with protease inhibitor (Cat# 11836145001, Roche, Basel, Switzerland) was added into a 2 mL homogenization tube containing 1.4 mm + 2.8 mm ceramic (zirconium oxide) beads (Cat# KT03961-1-009.2, Bertin, Aix-en-Provence, France). After loading the frozen sample, it was homogenized with a homogenizer (Precellys evolution homogenizer, Bertin, Aix-en-Provence, France) under the following settings: 5000 rpm for 4 × 30 s rounds with a 15-s break in between. Tubes were then centrifuged at 21,380× *g* at 4 °C for 5 min to allow the foams to set. Then, the tissue lysate was transferred into a new 1.5 mL Eppendorf tube and centrifuged for 30 min at 21,380× *g* at 4 °C. The lysate was then transferred into a new 1.5 mL Eppendorf tube and centrifuged for 15 min at 21,380× *g* at 4 °C. The protein concentration of vitreous tissue lysate was measured using the rapid gold BCA protein assay kit (Cat# A53225, Thermo Fisher Scientific, Waltham, MA, USA) for downstream sample preparation.

For reduction and alkylation, 0.1 M dithiothreitol (DTT) (Cat# 43815, Sigma–Aldrich, Burlington, MA, USA) was added to the sample to a final concentration of 10 mM and incubated at 37 °C for 1 h at 300 rpm. After that, 4 M iodoacetamide (IAA) (Cat# I1149, Sigma–Aldrich, USA) was added immediately to the sample at a final concentration of 40 mM and incubated at RT for 30 min in the dark. After alkylation, 100% ice-cold acetone was added to samples with a ratio of 1:4 (*v*/*v*) and kept at −20 °C overnight for acetone precipitation. Samples were centrifuged at 21,380× *g* for 30 min at 4 °C. The supernatant was discarded, and 500 µL of 80% acetone was added into the tubes and centrifuged again at 21380× *g* for 10 min at 4 °C. After drying, 10 µL of 4 M urea (CON_2_H_4_, Cat# 51456, Sigma–Aldrich, USA) in 25 mM ammonium bicarbonate (NH_4_HCO_3_, Cat# A6141, Sigma–Aldrich, USA) were added into each tube to dissolve the pellets. Thirty microliters of NH_4_HCO_3_ were added fold-wise to a final concentration of 1 M urea.

### 4.4. In-Solution Digestion and Sample Loading

Sequencing grade modified trypsin (Cat# V5111, Promega, Madison, WI, USA) was used throughout this study. A trypsin ratio of 1:25 (trypsin concentration: protein concentration *w*/*w*) was added to each sample for digestion for 16 h at 37 °C in the thermomixer (Eppendorf, Germany). Digestion was stopped by adding an appropriate amount of trifluoroacetic acid (TFA) to a final concentration of 0.1% TFA. Digested samples were then desalted and cleaned up with a C18 SPE HLB column (Waters, Framingham, MA, USA). Cleaned-up tryptic peptides were dried by vacuum centrifugation and were reconstituted in 0.1% formic acid. The peptide concentration was estimated using a peptide assay (Cat# 23275, Thermo Fisher Scientific, Waltham, MA, USA), and equal amounts of peptides from 6 samples were pooled together as OD and OS groups, resulting in an OD pool and OS pool for each time point. One microgram of digested vitreous peptides was loaded for MS injection.

### 4.5. LC-MS Acquisition

Both information-dependent acquisition (IDA) and data-independent acquisition (SWATH-MS) methods were performed on a TripleTOF^®^ 6600 quadrupole time-of-flight (QTOF) mass spectrometer (Sciex, Framingham, MA, USA) coupled to an Eksigent 415 nano-LC system (Sciex, USA). Equal amounts of digested peptides (according to each experiment condition settings) were loaded onto a C18 trap column (PepMap100, 5 μm, 100 Å, 100 μm i.d. × 20 mm, Thermo Fisher Scientific, USA) at a flow rate of 2 μL min^−1^ for 15 min and was then separated with a C18 nano-LC column (5 µm, 100 µm i.d. × 300 mm, Column Scientific, Xi’an, China) at a flow rate of 350 μL min^−1^ with a 10 μm SilicaTip electrospray emitter (New Objective Cat. No. FS360-20-10-N-20-C12). Mobile phase A was a mixture of 0.1% formic acid (*v*/*v*), 5% ACN (*v*/*v*) in water; mobile phase B contained 0.1% FA (*v*/*v*), 98% ACN (*v*/*v*) in water, with a linear gradient profile starting with 5% B and increasing to 45% B at 135 min. The linear gradient was increased to 80% from 135 to 141 min to clear off the possible hydrophobic peptides. The concentration of solve B was then dropped and kept to 5% for 10 min to prepare the system for the next injection. TOF-MS mass scan was set from 350 *m*/*z* to 1800 *m*/*z* with 250 ms accumulation time, followed by 100 *m*/*z*–1800 *m*/*z* for MS/MS scans in high sensitivity mode with 50 ms accumulation time of up to top 50 ion candidates per cycle, ions that exceed the threshold of 125 cps will be counted for MS/MS with the charge state between 2 and 4. Rolling collision energy was selected to trigger collision-induced dissociation. For data independent acquisition (DIA), the instrument was tuned for a variable isolation window in a looped mode over the mass range of 100 *m*/*z* to 1800 *m*/*z* scan of 100 overlapping variable windows. An accumulation time of 29 ms was set for each fragment ion, resulting in a total duty cycle of <3.0 s.

### 4.6. Study 1: Protein Identification and Quantification Using IDA and DIA (SWATH-MS)

Raw .wiff files of MS generated information dependent acquisition (IDA) were searched against the Gallus gallus UniProt database, and protein identification was acquired using ProteinPilot (v5.0, Sciex, USA). Search parameters were set as follows: trypsin as the enzyme, cysteine alkylation using iodoacetamide (IAA), thorough search effort, and biological modification were selected.

A 1% false discovery rate (FDR) was set as the filter for protein identification. For label-free quantification, a combined search of IDA injections was selected as the ion library for SWATH quantification. The IDA ion library and the SWATH injection files were loaded onto the SWATH Acquisition MicroApp 2.0 in PeakView (v2.2, Sciex, USA). Up to 10 peptides per protein, 6 transitions per peptide, 90% peptide confidence threshold, a 1% FDR, 10 min extracted-ion chromatogram (XIC) extraction window and 75 ppm width were selected for processing.

**Figure 11:**
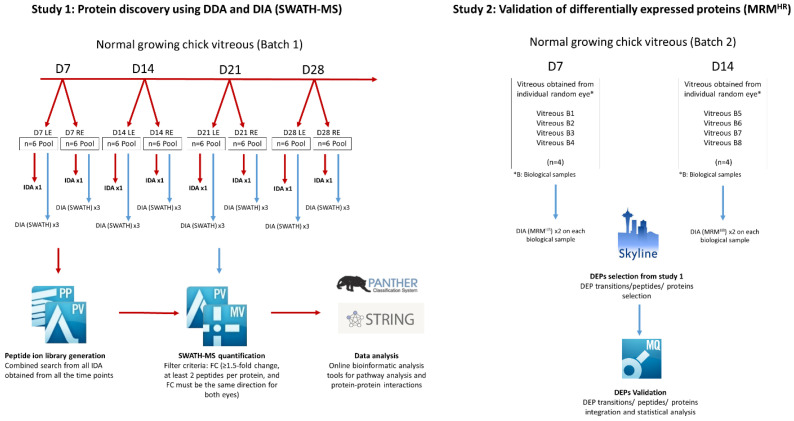
Schematic flowchart of the liquid chromatography–tandem mass spectrometry workflow with Study 1: discovery phase (IDA and SWATH-MS) and Study 2: Validation phase (MRM^HR^ validation).

Processed data were normalized using the most likely ratio (MLR) method [74] with MarkerView (v1.3, Sciex, USA) and exported to Excel for protein fold change calculation. Proteins with less than 1 peptide were removed to reduce the chances of false-positive results and the filter for differentially expressed proteins. A schematic flowchart of the liquid chromatography–tandem mass spectrometry workflow with the discovery phase (IDA and SWATH-MS) and validation phase (MRMHR validation) is shown in Figure 11.

### 4.7. Bioinformatic Analysis and Statistical Analysis

All values were presented as means ± standard deviation. The filter for differentially expressed proteins was considered at ≥ 1.5-fold change, which must be in the same direction for both eyes. The list of identified proteins was converted into gene names using the UniProt batch gene name tool online (http://www.uniprot.org/, accessed on 23 September 2023) [75]. A gene ontology (GO) analysis for functional classification biological processes, molecular functions, and cellular components was performed using the online analysis tool PANTHER (www.pantherdb.org) database version 14 [76]. The protein–protein interactions network analysis of significant differential expressed proteins was identified using STRING v12.0 (Search Tool for the Retrieval of Interacting Genes/Proteins) online database (http://www.string-db.org, accessed on 23 September 2023) [77]. The search engine was set as multiple proteins, and the list input was the accession numbers of the proteins; Gallus gallus was set as the organism. Protein–protein interactions were shown by colored lines.

### 4.8. Study 2: Validation by High-Resolution Multiple Reaction Monitoring (MRM^HR^)

A separate batch of chicks (*n* = 4 for each time point) was housed for 7 and 14 days in Study 2 under the same conditions as in Study 1. Refractive error and ocular measurements were measured to ensure the growth was similar to the normal growth study. The vitreous was collected and homogenized using a lysis buffer (T-PER) at a 1:1 *w*/*v* ratio. Equal amounts of vitreous proteins were taken out and samples were prepared (reduced, alkylated, digested, and cleaned-up) as previously reported. Two IDA injection replicates were combined and searched using ProteinPilot against the UniProt database (Gallus gallus). The list of differentiated proteins (DEPs) found across all the time points (D7, 14, 21, and 28) was loaded into the ion library, and a transition list of targeted peptides and MRM^HR^ acquisition method was created with Skyline Software [78]. A transition filter setting was further applied: 2, 3 precursor charges, 1 ion charge and y, b as the ion types. The ion match tolerance was set at 0.5 *m*/*z*, and the 5 most intense productions were selected. MRM^HR^ acquisitions were acquired using a TripleTOF^®^ 6600 quadrupole time-of-flight (QTOF) mass spectrometer (Sciex, USA). Equal amounts of digested (1.2 μg) samples were loaded onto a trap column (350 µm × 0.5 mm, C18) by loading the buffer (0.1% Formic acid, 2% Acetonitrile in water) at 2 µL min^−1^ for 15 min. It was then separated on a nano-LC column (100 µm × 30 cm, C18) using an Eksigent 415 nano-LC system at a flow rate of 350 nL min^−1^ with the following linear gradient starting with a linear gradient profile starting with 5% B and increasing to 45% B at 60 min. The linear gradient was increased to 80% from 65 to 75 min to clear off the possible hydrophobic peptides. The concentration of solve B was then dropped and kept at 5% for 10 min to prepare the system for the next injection. Peptides were injected into the mass spectrometer with a 10 μm SilicaTip electrospray emitter (New Objective Cat. No. FS360-20-10-N-20-C12). The DIA mode was acquired with the mass range of 100 *m*/*z* to 1800 *m*/*z* scan. An accumulation time of 29 ms was set for each fragment ion, resulting in a total duty cycle of 3.0 s. MultiQuant (v3.0, Sciex, Framingham, MA, USA) was used to integrate the areas of the transition ions for each peptide. Only transitions that had a signal-to-noise ratio above 20 were selected. The top 3 peptides with the most intensity transition ions were selected and normalized with GAPDH for fold change calculation (day 14/day 7). Top peptides were averaged, and the FC was calculated (D14/D7). Unpaired *t*-tests were performed between vitreous samples collected from individual animals (*n* = 4 at each time point) for relative quantification between D14 and D7 samples, with a *p*-value less than 0.05, to be considered statistically significant.

## 5. Conclusions

This is the first study investigating the proteomic changes in the vitreous humor of chicks during normal growth using next-generation mass spectrometry techniques for identification, quantitation, and validation (SWATH-MS and MRMHR). The findings of this study have provided a comprehensive spectral library of the vitreous during normal growth, which could serve as the foundation for future proteomic studies on the vitreous. The utilization of the SWATH-MS enables a more in-depth identification and quantification of vitreous proteins, greatly enhancing the chances of the detection of differentially expressed proteins. The comprehensive proteome acquired from this study further refines the knowledge on the small portion of proteins that are within the vitreous, even though they are known to be mainly composed of water. Quantitative proteomics of the vitreous (SWATH-MS) during various time points in the emmetropization period unraveled growth-related structural proteins, such as AFP, TF, NCAM and CDH, showed that the vitreous humor could be a compartment to reflect changes in growth, which could contribute to the natural eye growth process, resulting in the elongation of the eyeball. The MS peptide raw data from the IDA and SWATH injections have been deposited to the Peptide Atlas (www.peptideatlas.org/PASS/PASS01258, accessed on 27 September 2019). Raw data from the targeted validation using MRM^HR^ have been deposited to the Proteomics Identifications database https://www.ebi.ac.uk/pride/archive/projects/PXD056184, accessed on 25 September 2024).

## Figures and Tables

**Table 1:** Growth-related differentially expressed proteins (DEPs) found across all time points (days 14, 21, and 28). The fold change (FC) was calculated against day 7, FC cut off ≥1.5 or ≤0.7, and both eyes must be in the same FC direction. Red: up-regulated proteins, Blue: down-regulated proteins.

			(Day 14/7) SWATH-MS FC			(Day 21/7) SWATH-MS FC			(Day 28/7) SWATH-MS FC		
UniProt Accession Number	Protein Name	Gene Name	OD14/OD7	OS14/OS7	AVE FC	OD21/OD7	OS21/OS7	AVE FC	OD28/OD7	OS28/OS7	AVE FC
R4GLH0	IGFBP N-terminal domain-containing protein	ESM1	4.08	3.02	3.55 ± 0.75	4.53	3.51	4.02 ± 0.72	6.73	5.09	5.91 ± 1.16
Q4ADJ6	Ovotransferrin	TFEW	4.09	2.3	3.2 ± 1.27	1.81	2.26	2.04 ± 0.32	9.39	5.11	7.25 ± 3.03
P84407	Alpha-fetoprotein	AFP	0.01	0.4	0.21 ± 0.28	0.01	0.01	0.01 ± 0.00	0.01	0.02	0.02 ± 0.01
P79995	Cadherin-10	CDH10	0.46	0.12	0.29 ± 0.24	0.43	0.39	0.41 ± 0.03	0.4	0.35	0.38 ± 0.04
P24503	Cadherin-4	CDH4	0.31	0.32	0.32 ± 0.01	0.34	0.43	0.39 ± 0.06	0.37	0.4	0.39 ± 0.02
E1C3A7	Cadherin 22	CDH22	0.41	0.14	0.28 ± 0.19	0.34	0.41	0.38 ± 0.05	0.33	0.3	0.32 ± 0.02
Q8AWW2	Cadherin-7	N/A	0.33	0.38	0.36 ± 0.04	0.39	0.32	0.36 ± 0.05	0.32	0.34	0.33 ± 0.01
Q8QGH3	Cadherin-20	CDH20	0.47	0.61	0.54 ± 0.1	0.44	0.44	0.44 ± 0.00	0.45	0.53	0.49 ± 0.06
Q9W6E1	Neurocan core protein	N/A	0.23	0.48	0.36 ± 0.18	0.21	0.2	0.21 ± 0.01	0.17	0.19	0.18 ± 0.01
Q90953	Versican core protein	VCAN CSPG2	0.33	0.59	0.46 ± 0.18	0.2	0.25	0.23 ± 0.04	0.19	0.19	0.19 ± 0.00
O93574	Reelin	RELN	0.41	0.58	0.5 ± 0.12	0.45	0.48	0.47 ± 0.02	0.42	0.47	0.45 ± 0.04
F1NSJ1	Contactin-2	CNTN2	0.51	0.23	0.37 ± 0.2	0.44	0.46	0.45 ± 0.01	0.52	0.52	0.52 ± 0.00
P35331	Neuronal cell adhesion molecule (Nr-CAM)	NRCAM	0.32	0.57	0.45 ± 0.18	0.59	0.56	0.58 ± 0.02	0.67	0.5	0.59 ± 0.12
Q90610	Neogenin (Fragment)	NEO1	0.65	0.39	0.52 ± 0.18	0.54	0.55	0.55 ± 0.00	0.67	0.62	0.65 ± 0.04

## Data Availability

The MS peptide raw data from the IDA and SWATH injections have been deposited to the Peptide Atlas (https://www.peptideatlas.org/PASS/PASS01258, accessed on 27 September 2019). The raw data of targeted validation using MRMHR have been deposited to the Proteomics Identifications database (https://www.ebi.ac.uk/pride/archive/projects/PXD056184, accessed on 25 September 2024). The data presented in this study are available upon request from the corresponding author.

## Supplementary Materials

The following supporting information can be downloaded at: https://www.mdpi.com/article/10.3390/ijms251910644/s1.
